# Supplementary material for: Meningeal inflammation in multiple sclerosis induces phenotypic changes in cortical microglia that differentially associate with neurodegeneration
Source: Acta Neuropathol. 2021 Mar 29;141(6):881–99. doi: 10.1007/s00401-021-02293-4 (PMC8113309; doi:10.1007/s00401-021-02293-4)
Supplement: Supplementary file 1 — Supplementary file1 (PDF 1086 KB) [file 401_2021_2293_MOESM1_ESM.pdf]

Online Resource Material

Meningeal inflammation in multiple sclerosis induces phenotypic changes in cortical microglia that differentially associate with neurodegeneration

Lynn van Olst[1#], Carla Rodriguez-Mogeda[1#], Carmen Picon-Munoz[2], Svenja Kiljan[3], Rachel E. James[2], Alwin Kamermans[1], Susanne M.A. van der Pol[1], Lydian Knoop[4], Iliana Michailidou[5], Evelien Drost[1], Marc Franssen[1], Geert J. Schenk[3], Jeroen J.G. Geurts[3], Sandra Amor[4], Nicholas D. Mazarakis[2], Jack van Horssen[1], Helga E. de Vries[1,6], Richard Reynolds[2,5]* & Maarten E. Witte[1,4]*

[1] Department of Molecular Cell Biology and Immunology, Amsterdam UMC, MS Center Amsterdam, Amsterdam Neuroscience, Amsterdam, Netherlands

[2] Division of Neuroscience, Department of Brain Sciences, Imperial College London, Hammersmith Hospital Campus, Burlington Danes Building, Du Cane Road, London W12 0NN, UK

[3] Department of Anatomy & Neurosciences, Amsterdam UMC, MS Center Amsterdam, Amsterdam Neuroscience, Amsterdam, Netherlands

[4] Department of Pathology, Amsterdam UMC, MS Center Amsterdam, Amsterdam Neuroscience, Amsterdam, Netherlands

[5] Department of Clinical Genetics, Leiden University Medical Center, Leiden, Netherlands

[6] Department of Medical Biochemistry, Amsterdam UMC, Amsterdam Cardiovascular Sciences, Amsterdam, The Netherlands

[7] Centre for Molecular Neuropathology, LKC School of Medicine, Nanyang Technological University, Singapore, Singapore

[#] Both authors contributed equally to this work

* These authors jointly supervised this work: Correspondence and requests for materials should be addressed to M.E.W. (email: m.e.witte@amsterdamumc.nl, tel: +31(0) 20 44 48080) or R.R. (email: r.reynolds@imperial.ac.uk).

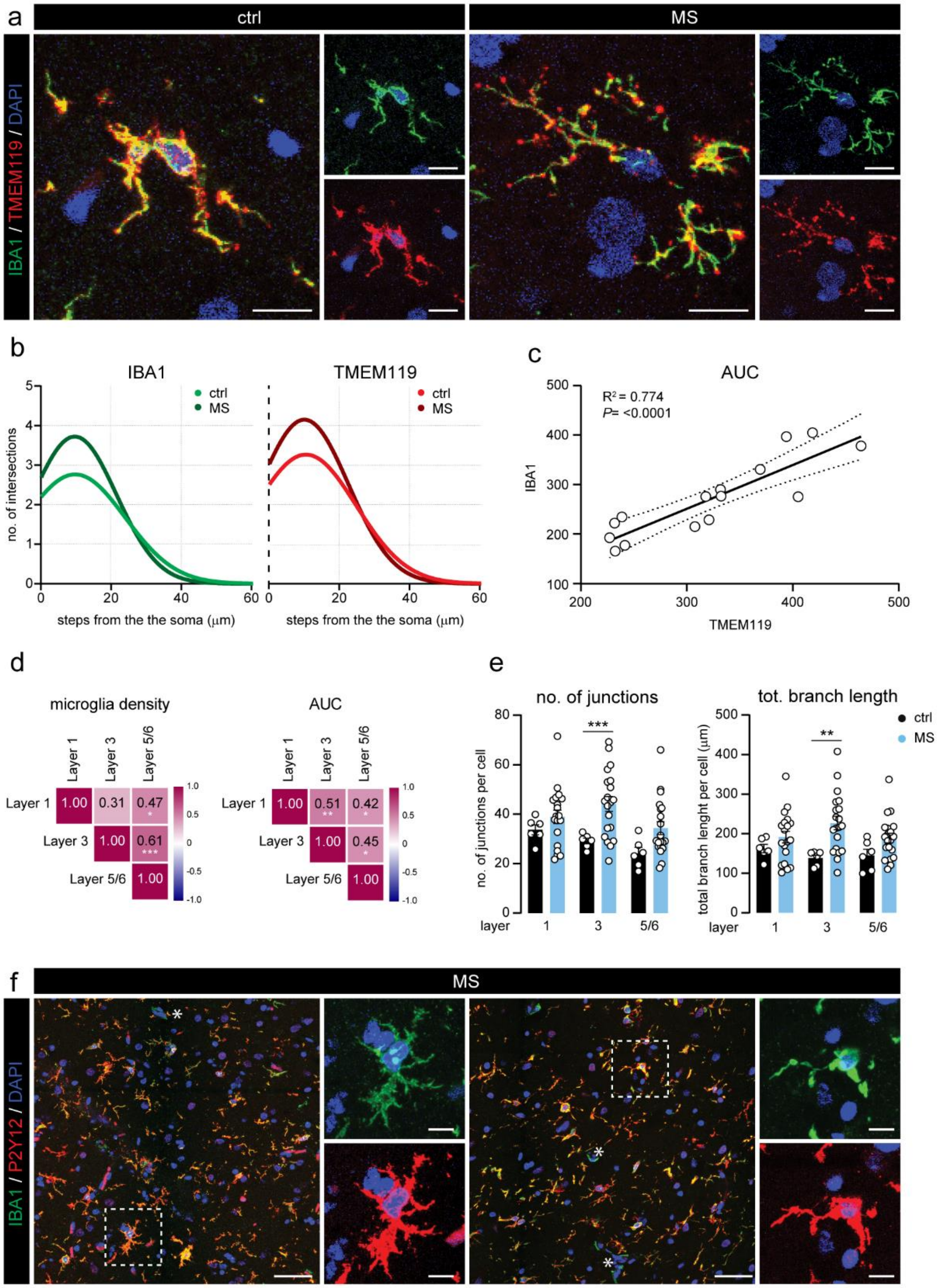

Online Resource 1

a. Micrographs of single IBA1$^+$, TMEM119$^+$ cortical microglia in layer 3 from a ctrl and MS donor. **b**. Non-linear curve fit of the average number of microglial branch intersections per 0.3 µm step from the cell soma

per cortical layer of $IBA1^+$ and $TMEM119^+$ cells as measured by Sholl analysis. **c**. Quantification of the correlation between the total Sholl-derived area-under-the-curve (AUC) of $IBA1^+$ and $TMEM119^+$ cells. **d.** Correlation matrix (with *r* correlation coefficients depicted within the squares) of microglia density and shape (AUC) between the different neuronal layers. **e**. Quantification of the number of junctions and total branch length of microglia in the different neuronal layers of microglial cell morphology. **f**. Representative image of P2Y12 and IBA1 co-expression in cortical layer 3 of two MS subjects. Asterisks depict $IBA1^+$ vessel-associated cells that are negative for P2Y12 expression. Individual datapoints indicate averaged data from an individual donor, columns and error bars show mean ± SEM; *p < 0.05, **p < 0.01, *** p < 0.001; *n* = 6 ctrl and *n* = 9 MS (**b**, **c**), *n* = 6 ctrl and *n* = 20 MS (**e**); Scale bars = 20 µm (**a)**, 25 µm (**f;** overview), 10 µm (**f**: close-up).

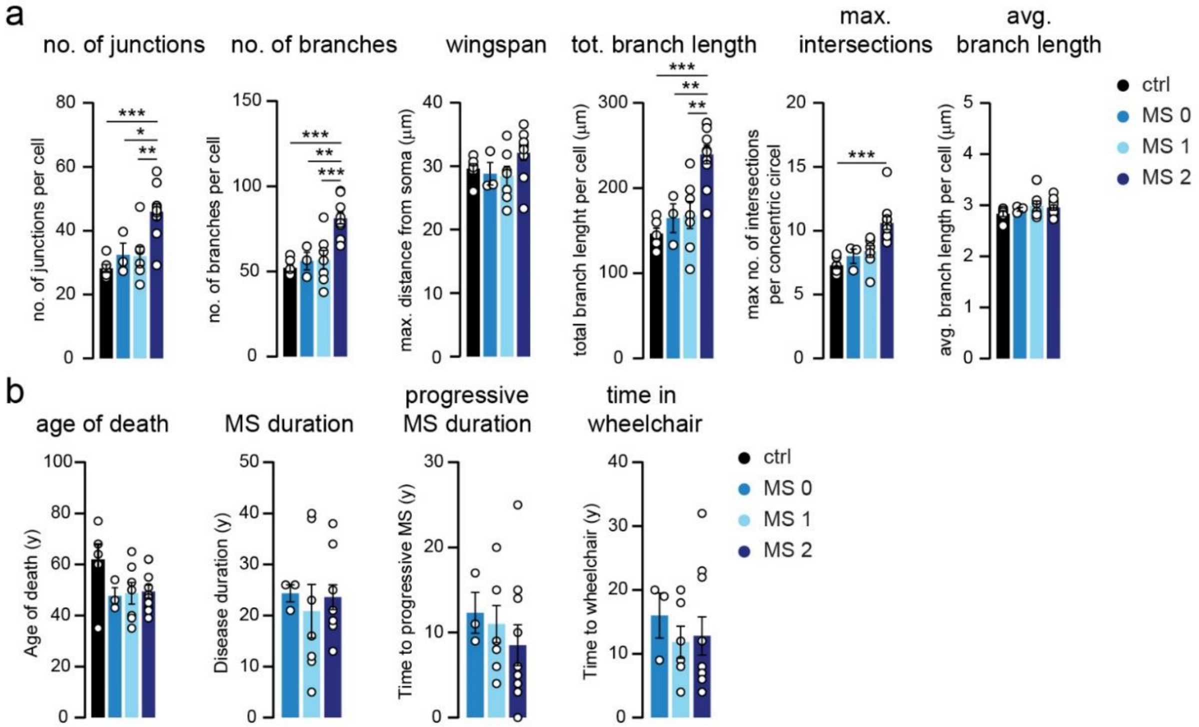

Online Resource 2

a. Quantification of the average number of junctions, number of branches, wingspan, total branch length, maximum number of intersections and average branch length of microglial cell morphology in the different MS sub-clusters as detected by K-means clustering. **b.** Quantification of the age of death, MS disease duration, progressive MS duration and time in wheelchair bound in the different MS sub-clusters as detected by K-means clustering. Individual datapoints indicate averaged data from an individual donor, columns and error bars show mean ± SEM; *p < 0.05, **p < 0.01, *** p < 0.001; *n* = 6 ctrl, *n* = 3 MS0, *n* = 7 MS1, *n* = 10 MS2.

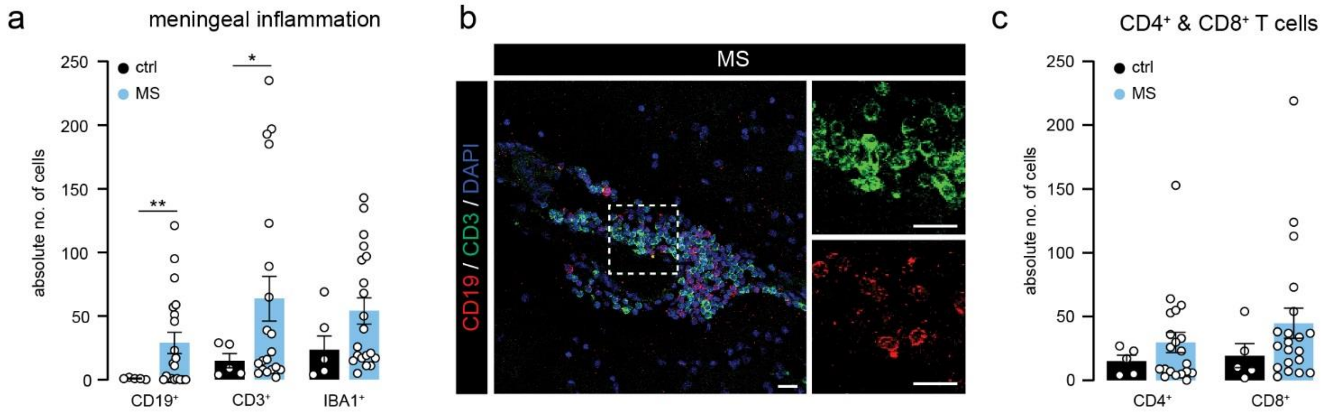

Online Resource 3

a. Quantification of the absolute cell count of CD19^{+} B cells, CD3^{+} T cells and IBA1^{+} myeloid cells in the meninges of ctrl and MS cases. **b**. Representative images of a cluster of CD19^{+} B cells and CD3^{+} T cells in meninges of a MS subject. **c**. Quantification of the absolute cell count of CD4^{+} and CD8^{+} T cells in the meninges of ctrl and MS subjects. Individual datapoints indicate averaged data from an individual donor, columns and error bars show mean ± SEM; *p < 0.05, **p < 0.01; *n* = 5 ctrl, *n* = 20 MS; Scale bars = 20 µm.

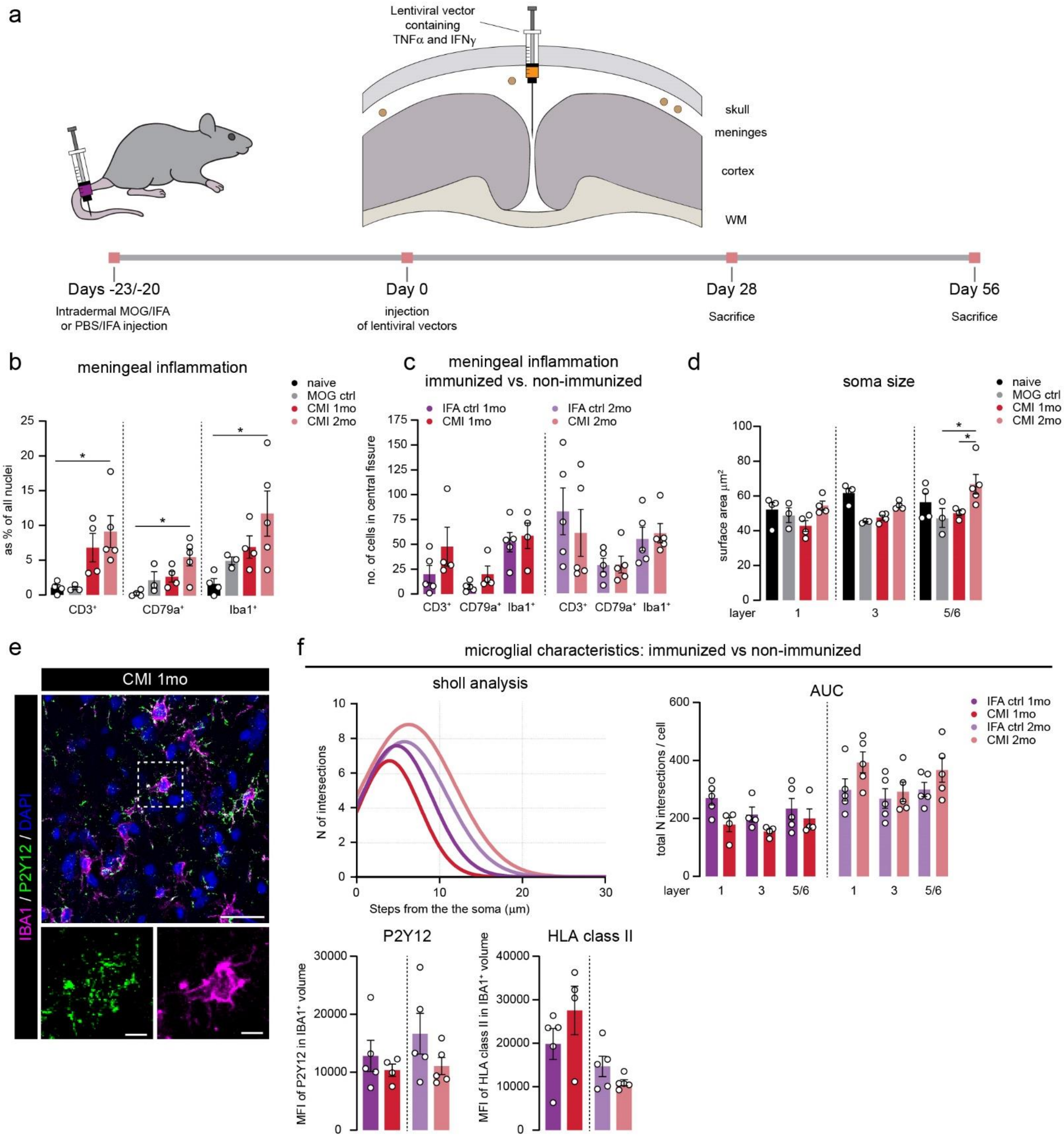

Online Resource 4

a. Experimental setup of chronically induced meningeal inflammation in rats. Animals were either immunized with MOG/IFA (CMI) or injected with PBS/IFA (IFA ctrl) 23/20 days before injection of lentiviral constructs carrying the TNFα and IFNγ genes vectors in the sagittal sulcus. Animals were

sacrificed at day 28 (1 month) or day 56 (2 months). **b.** Quantification of $CD3^+$ T cells, $CD79a^+$ B cells and $IBA1^+$ myeloid cells as percentage of all extravascular nuclei in the sagittal sulcus of naive, MOG ctrl, CMI 1 month and CMI 2 month animals. **c**. Absolute number of meningeal $CD3^+$, $CD79a^+$ and $IBA1^+$ cells in immunized (CMI) vs. non-immunized (IFA ctrl) rats. **d.** Quantification of the microglial soma size in layer 1, 3 and 5/6 of the cortex from naive, MOG ctrl, CMI 1 month and CMI 2 month animals. **e.** Representative image of cortical layer 3 of a CMI 1 month rat with the highest microglial density immunostained for IBA1 and P2Y12. **f**. Different measurements of $IBA1^+$ microglia: Non-linear curve fit of the average number of microglial branch intersections per 0.3 µm step from the cell soma as measured by Sholl analysis; total Sholl-derived area-under-the-curve (AUC); mean fluorescence intensity of P2Y12 and HLA class II in immunized (CMI) vs. non-immunized (IFA ctrl) rats. Individual datapoints indicate averaged data from an individual donor, columns and error bars show mean ± SEM; $^*p < 0.05$, $^{**}p < 0.01$, $^{***} p < 0.001$; $n = 4$ naïve, $n = 3$ MOG ctrl, $n = 5$ IFA ctrl 1mo, $n = 4$ CMI 1mo, $n = 5$ IFA ctrl 2mo, $n = 5$ CMI 2mo (**b**, **c**, **e**, **f**). Scale bars = 25 µm (**d**: overview); 5 µm (**d**: close-up).

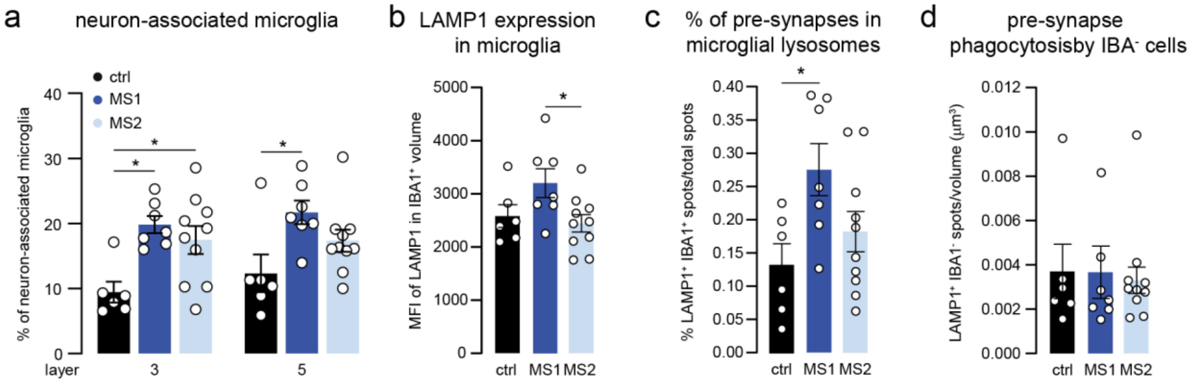

Online Resource 5

a. Percentage of microglial cell bodies that are directly in contact with neuronal somata in cortical layers 3 and 5/6 of ctrl, MS1 and MS2. **b.** Quantification of the mean fluorescence intensity of LAMP1 in IBA1$^+$ volume in cortical layer 3 of ctrl, MS1 and MS2 donors. **c.** Percentage of all Synaptophysin$^+$ pre-synapses that are located in microglial lysosomes in cortical layer 3 of ctrl, MS1 and MS2. **d.** Density of pre-synapses found within lysosomes of IBA1$^-$ cells in layer 3 of ctrl, MS1 and MS2 cases. Individual datapoints indicate averaged data from an individual donor, columns and error bars show mean ± SEM; *$p < 0.05$; n = 6 ctrl, n = 7 MS1, n = 10 MS2.

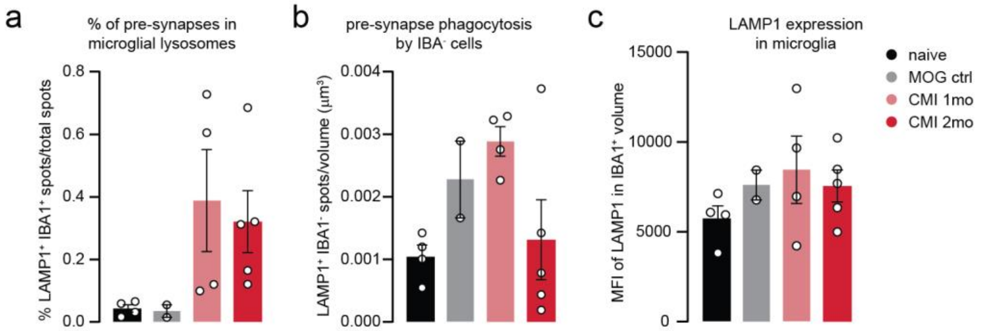

Online Resource 6

a. Percentage of all vGAT^{+} pre-synapses that are located in microglial lysosomes in cortical layer 3 of naive, MOG ctrl, CMI 1 month and CMI 2 month animals. **b.** Density of pre-synapses found within lysosomes of IBA1^{-} cells in layer 3 of naive, MOG ctrl, CMI 1 month and CMI 2 month animals. **c.** Quantification of the mean fluorescence intensity of LAMP1 in cortical layer 3 microglia in naive, MOG ctrl, CMI 1 month and CMI 2 month animals. Individual datapoints indicate averaged data from an individual animal, columns and error bars show mean ± SEM; *n* = 4 naïve, *n* = 2 MOG ctrl, *n* = 4 CMI 1mo, *n* = 5 CMI 2mo.
